# Sulfonic acid-functionalized chitosan–metal–organic framework composite for efficient and rapid conversion of fructose to 5-hydroxymethylfurfural

**DOI:** 10.1038/s41598-024-56592-3

**Published:** 2024-03-10

**Authors:** Sima Darvishi, Samahe Sadjadi, Majid M. Heravi

**Affiliations:** 1 Department of Chemistry, School of Physics and Chemistry, Alzahra University, PO Box 1993891176, Vanak, Tehran, Iran; 2 Gas Conversion Department, Faculty of Petrochemicals, Iran Polymer and Petrochemical Institute, PO Box 14975-112, Tehran, Iran

**Keywords:** Heterogeneous catalysis, Chemistry

## Abstract

In pursuit of designing a bio-based catalyst for the dehydration of biomass (i.e., fructose) to 5-hydroxymethylfurfural, a novel catalytic composite was prepared by in-situ formation of an Al-based metal–organic framework in the presence of chitosan. To enhance the acidity of the as-prepared catalyst, it was sulfonated with chlorosulfonic acid. Various characterization techniques, including XRD, XPS, FTIR, SEM/EDX, TGA, and elemental mapping analysis were applied to validate the formation of the acidic composite. Fructose dehydration conditions were also optimized using Response Surface Method (RSM) and it was found that reaction in the presence of catalyst (23 wt%) in DMSO, at 110 °C for 40 min led to the formation of HMF in 97.1%. Noteworthy, the catalyst was recyclable and stable up to five runs with a minor reduction in its activity.

## Introduction

Increasing environmental concerns, such as global warming and pollutants in the atmosphere and demand for sustainable development^1–3^ resulted in the expansion of use of renewable energies. In this venue, the conversion of biomass to value-added products and fuels garnered growing attention^4–7^. One of the key chemicals that can be obtained from the conversion of biomass is 5-hydroxymethyl-2-furfural (HMF)^8^ which offers a wide range of applications in chemical and petrochemical industries^9–11^. This furan-based compound is also the precursor for the production of 2,5-dimethylfuran, which is deemed a potential biofuel with comparable energy density to conventional fossil fuels and a high-value-added bioplastic monomer 2,5-furandicarboxylic acid (FDCA)/2,5-bis(hydroxymethyl)furan (BHMF)^12–20^. These features triggered extensive research on the efficient synthesis of HMF. Mainly, HMF is achieved through the conversion of mono, de, and polysaccharides under acidic conditions^21,22^. Among the available precursors, fructose is frequently selected for HMF synthesis due to its abundant availability and cost-effectiveness^23^. The main challenge in this reaction is the formation of some by-products and/or condensation of HMF to humin^24^. In this regard, design of an efficient and low-cost catalyst that can promote this reaction with high selectivity to HMF is of great importance^25–27^.

The acidic composite based on metal–organic framework (MOF) has gained significant attention as an efficient catalyst^28–31^. By combining the unique properties of biopolymers with the highly porous structure and tunable acidity of MOFs, researchers have achieved catalysts with enhanced catalytic efficiency, stability, and eco-friendly characteristics^32–34^. The previous studies highlight the importance of optimizing the synthesis parameters, investigating the reaction conditions, and understanding the underlying mechanisms to further improve the performance of this bio-based catalyst^35–37^. One of the most appealing classes of compounds that can be applied for catalysis is chitosan (CS)^38–40^, which is a biodegradable and biocompatible naturally occurring carbohydrate. As CS surface is rich in functional groups, it can be readily functionalized or incorporated into the structure of composites in covalent and non-covalent approaches. As an instance, CS has been conjugated with MOFs to furnish composite^41^, which benefits from the characteristics of both CS and MOFs, which are porous crystalline materials with tunable porosity^42–44^. Interestingly, the acidic features of both CS and MOF can be adjusted by several approaches, such as functionalization^45,46^. This approach provides a potential solution to the relatively low acidity of unmodified MOFs and carbohydrates and offers a pathway for designing task-specific catalysts. On the other hand, the application of the acidic composite of CS and MOF in fructose dehydration represents a significant step towards sustainable and environmentally friendly catalytic processes in the field of chemical engineering.

**Figure 1:**
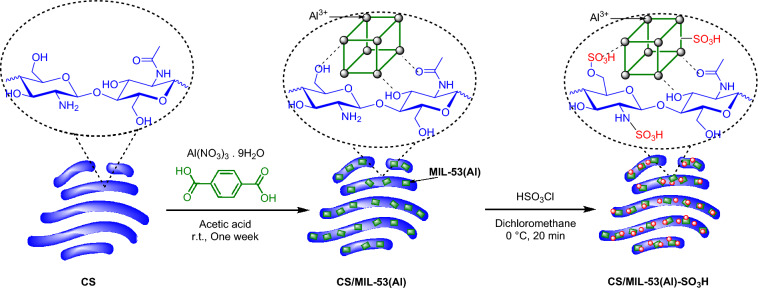
Pictorial procedure for the preparation of CS/MIL-53(Al)-SO_3_H.

In pursuit of our research on disclosing novel catalysts for HMF synthesis^27,47,48^, in this study with the aim of CS and MOF chemistry combination, a novel composite is formed by in-situ formation of Al-based MOF in the presence of CS (Fig. 1). To enhance the acidity of the resultant composite, it was sulfonated to furnish CS/MIL-53(Al)-SO_3_H, which was utilized as an acidic catalyst for dehydration of fructose to HMF. We hypothesized that CS serves as a support matrix for the MOF and provides structural stability, preventing their leaching during the reaction and enabling the composite to be reused multiple times. Additionally, CS contains amino functional groups that participated in sulfonating reaction. Sulfonic acid (-SO_3_H)-functionalized CS can further enhance the catalytic activity of the composite. On the other hand, CS/MOF composite combines the advantageous properties of chitosan and MOF (extra acid functionality, high surface area and stability) to create an efficient system for the conversion of fructose to HMF. By understanding the roles of each component, we can appreciate the importance of this composite material in the field of sustainable chemistry and biomass conversion. The influential reaction parameters were optimized using the Response Surface Method (RSM) and recyclability and catalytic activity of the catalyst were appraised and compared with un-sulfonated counterpart. Additionally, plausible reaction mechanism was proposed.

## Result and discussion

### Characterization of the synthesized catalyst

**Figure 2:**
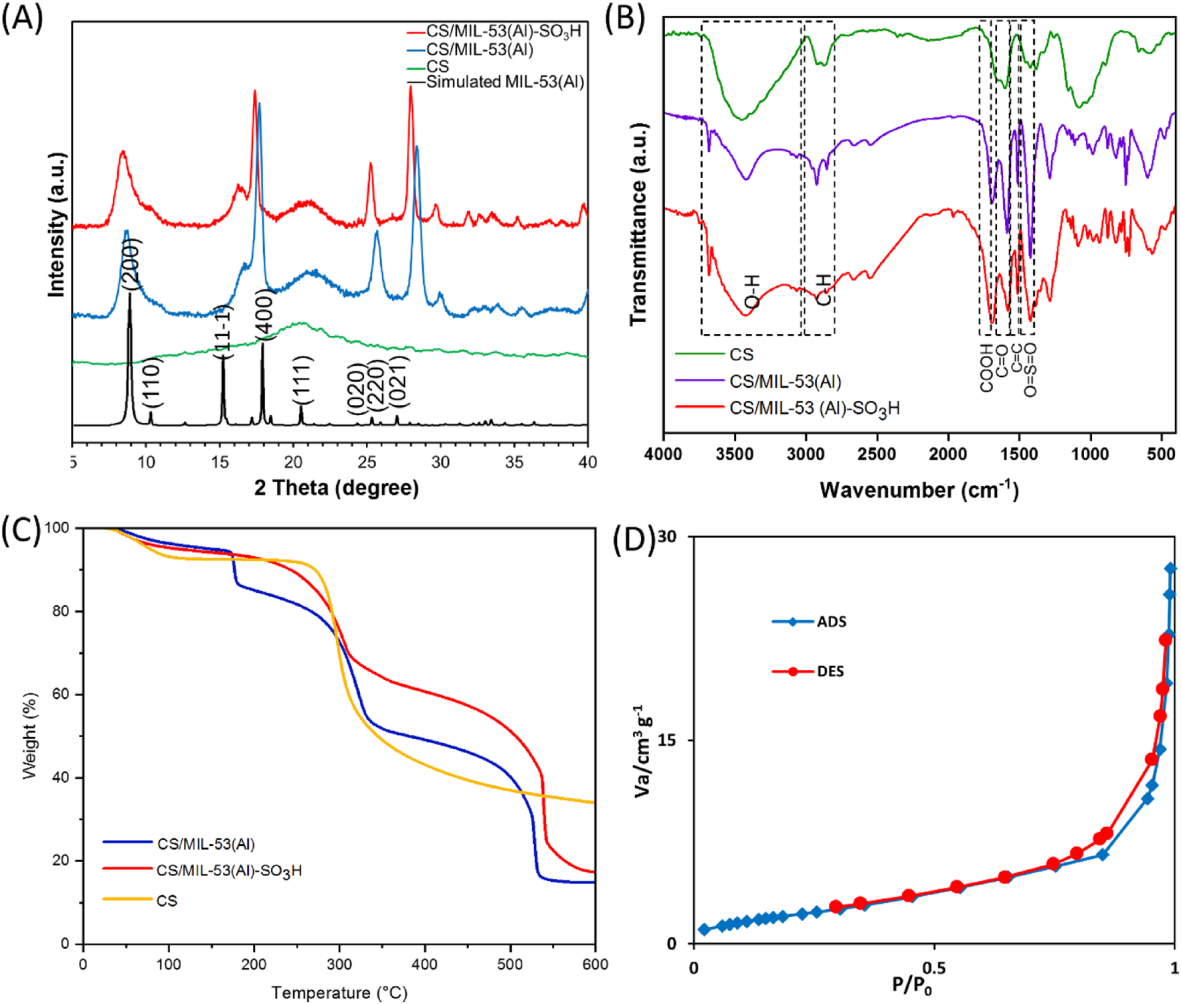
(**A**) XRD patterns and (**B**) FTIR spectra of CS, CS/MIL-53(Al) and CS/MIL-53(Al)-SO_3_H. (**C**) TG curves of CS/MIL-53(Al) and CS/MIL-53(Al)-SO_3_H CS. (**D**) Nitrogen adsorption–desorption isotherm of CS/MIL-53(Al)-SO_3_H at 77 K. The simulated pattern for the MIL-53(Al), calculated from the CIF file with Mercury software has also been included for comparison reported by Loiseau et al^56^.

To study the structure and crystalline phase of CS/MIL-53(Al)-SO_3_H it was subjected to XRD analysis and its XRD pattern was compared with that of CS and CS/MIL-53(Al). As anticipated, in the XRD pattern of CS, which is an amorphous carbohydrate a broad peak in the range of 2θ = 13–30° is detected, Fig. 2A. In the XRD pattern of CS/MIL-53(Al), the broad peak that is indicative of CS is observable. Furthermore, the assigned characteristic peaks at 2θ = 9°, 10.1°, 15.6°, 17.2°, 21.2, 25°, 26.1°, and 27.4° corresponding to the (200), (110), (11–1), (400), (111), (020), (220), and (021) planes of MIL-53(Al), respectively, confirm the formation of MIL-53(Al) in the presence of CS^49^. The XRD pattern of CS/MIL-53(Al)-SO_3_H is similar to that of CS/MIL-53(Al) and exhibited the characteristic bands of both CS and CS/MIL-53(Al). This outcome provides evidence that sulfonation of CS/MIL-53(Al) did not cause structural change and the crystalline phase of CS/MIL-53(Al) was preserved.

To offer insight into the structure of CS/MIL-53(Al)-SO_3_H and verify its formation, FTIR spectrum of the as-prepared catalyst was compared with that of CS and CS/MIL-53(Al), Fig. 2B. According to the literature ^50^, the absorbance bands at 3479 cm^−1^, 2908 cm^−1^, 1627 cm^−1^ in the FTIR spectrum of CS are attributed to the –OH, -CH_2_ and C = O functionalities respectively. FTIR spectrum of CS/MIL-53(Al) is distinguished from CS and exhibited some additional bands. More accurately, the bands at 1481 cm^−1^, 1589 cm^−1^, 1703 cm^−1^ are attributed to –COOH functionality^51^, while the band that appeared at 1514 cm^−1^ is representative of –C = C group. FTIR spectrum of CS/MIL-53(Al)-SO_3_H exhibited the characteristic bands of CS/MIL-53(Al), emphasizing that CS/MIL-53(Al) was structurally stable upon sulfonation. The broadening of some bands in the range of 1417–1487 cm^−1^ is ascribed to the presence of O = S = O stretching bonds^52^.

TG curves of CS/MIL-53(Al), CS/MIL-53(Al)-SO_3_H and CS are presented in Fig. 2C. The TG curve of CS is aligned with the previous reports, encompassing the weight loss due to the removal of water and CS decomposition (330 °C). CS/MIL-53(Al) TG curve exhibited three weight loss steps at 150, 330 and 540 °C, which are due to the water evaporation, CS and MIL-53(Al) decomposition respectively. Comparison of thermograms of CS/MIL-53(Al) and CS/MIL-53(Al)-SO_3_H supported that sulfonation resulted in increase of the thermal stability of CS/MIL-53(Al).

In order to assess the porous structure of the synthesized CS/MIL-53(Al)-SO_3_H, nitrogen adsorption–desorption analysis was conducted at a temperature of 77 K. The obtained isotherm displayed characteristics that are typical of nanoporous materials (Fig. 2D). These characteristics are aligned with Type III classification as defined by the International Union of Pure and Applied Chemistry (IUPAC)^53^. Besides, the total pore volume, BET surface area, and mean pore diameter were measured to be 0.04 cm^3^/g, 8.0209 m^2^/g, and 20.411 nm for CS/MIL-53(Al)-SO_3_H, respectively. Some works reported similar results, lower surface area compared to pristine MOF, for in-situ forming MOF in the biopolymeric material^54,55^. Performing this analysis gains further insight into the intricate porous nature of the CS/MIL-53(Al)-SO_3_H compound, thereby its suitable potential applications as catalysts.

**Figure 3:**
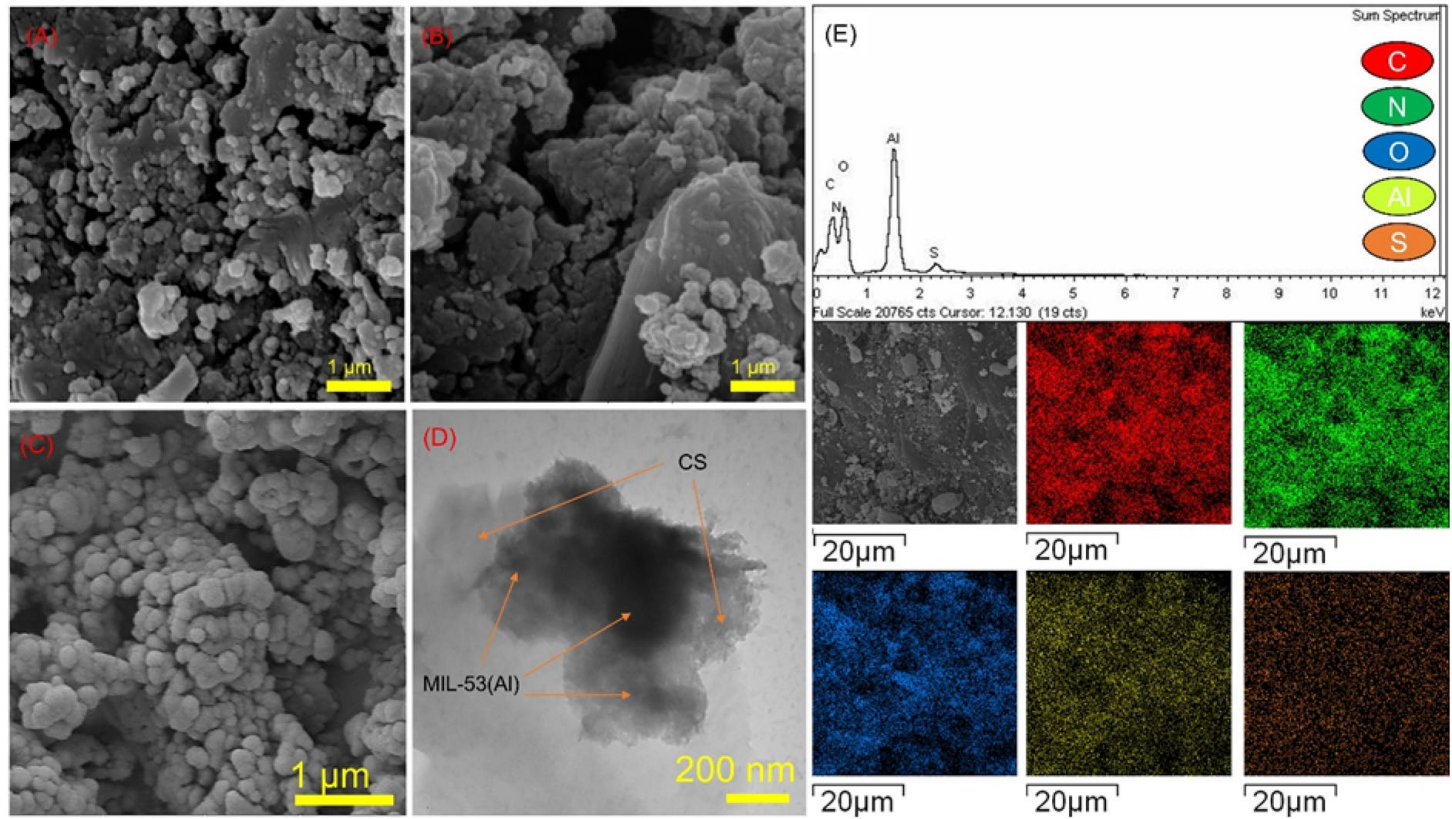
(**A**) SEM image of CS/MIL-53(Al), (**B**) CS/MIL-53(Al)-SO_3_H catalyst, (**C**) MIL-53(Al), (**D**) TEM image of CS/MIL-53(Al)-SO_3_H catalyst and E) EDX/Mapping images of CS/MIL-53(Al)-SO_3_H.

The morphology of MIL-53(Al), CS/MIL-53(Al) and CS/MIL-53(Al)-SO_3_H was unveiled via SEM, Fig. 3A-C. MIL-53(Al) sample is morphologically homogeneous and displays agglomerates/aggregates of nanocrystals within the range 20–100 nm^57^. As depicted in the SEM image of CS/MIL-53(Al), small particles are interconnected within CS body. The morphology of CS/MIL-53(Al)-SO_3_H demonstrates a deviation from the original CS/MIL-53(Al) structure. The presence of MIL-53(Al) framework within the CS polymeric structure was studied by TEM analysis. As illustrated in Fig. 3D, MIL-53(Al) crystals have appeared as spots dispersed over CS bodies. This observation highlights the fact that the process of sulfonation can induce a certain level of morphological alteration.

To further characterize CS/MIL-53(Al)-SO_3_H, EDX and elemental mapping analyses were carried out. According to the results, shown in Fig. 3E, the a-prepared nanocomposite contains N, O, C, Al and S atoms in its structure. Among the detected atoms, Al, C and O can signify MIL-53(Al), while C, O and N are attributed to CS structure. Additionally, the presence of S atom underlines the successful sulfonation of CS/MIL-53(Al) nanocomposite. Elemental mapping analysis of CS/MIL-53(Al)-SO_3_H emphasizes that sulfonation of the as-prepared nanocomposite was conducted uniformly and it can be deduced and –SO_3_H functionality was provided throughout CS/MIL-53(Al).

**Figure 4:**
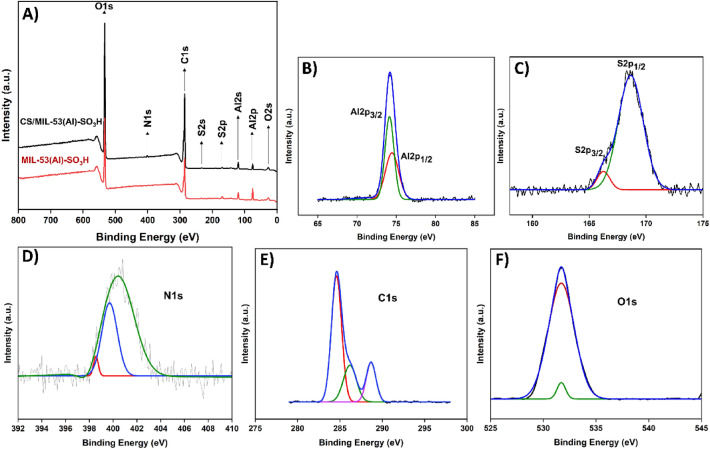
Survey XPS (**A**), high-resolution Al (**B**), high-resolution S (**C**), high-resolution N (**D**), and high-resolution C (**C**), high-resolution O (F) spectra of CS/MIL-53(Al)-SO_3_H and MIL-53(Al)-SO_3_H.

XPS analysis as a potent characterization technique was also utilized for exploring the catalyst. The presence of the characteristic peaks of aluminum (Al 2p at 75 eV), oxygen (O1s at 552 eV), (S 2p at 178 eV), carbon (C1s at 285 eV), in the full scan XPS spectra of MIL-53(Al)-SO_3_H, confirmed the successful functionalization of MIL-53(Al) with -SO_3_H. Besides, comparison of the Survey XPS patterns of MIL-53(Al)-SO_3_H and CS/MIL-53(Al)-SO_3_H indicated an additional nitrogen peak (N1s at 401 eV), demonstrating the presence of CS in structure of the designed catalyst, CS/MIL-53(Al)-SO_3_H, Fig. 4A. High-resolution Al 2p profile, Fig. 4B, was deconvoluted into the peaks at, 74.7 eV and 74.8 eV, which are representative of Al species in MIL-53(Al)^58–60^. Deconvolution of S 2p spectrum resulted in two peaks at 168.2 and 169.2 eV that are assigned to S–O and S = O bonds (Fig. 4C)^61,62^. According to the deconvoluted high-resolution N1s spectrum, Fig. 4D, peaks at 398.7, 399.8, and 400.9 eV can be observed related to an amine (C–NH_2_), amide (C–NHC = O) and protonated amine (C–N^+^), respectively^63^. The C1s spectrum can also be deconvoluted to the characteristic peaks at 289.2 eV, 286.2 eV and 284 eV, Fig. 4E, which are indicative of C atoms in benzene ring and, as well as carboxylate in MIL-53(Al) and carbon species in CS. As seen, in the high-resolution O 1s the peaks at 530.4 eV and 532 eV, were detected Fig. 4F, which are ascribed to carboxylate group and C-O bonds^64^.

### Control catalysts

It is well-known that dehydration of fructose to HMF is promoted by acidic catalysts. Hence, the as-prepared CS/MIL-53(Al) was sulfonated to provide more acidic active sites on the catalyst and improve its catalytic performance. Using NH_3_-TPD (Fig. S5), the acidity characteristic CS/MIL-53(Al)-SO_3_H was also investigated. According to the results, the catalyst possesses strong acidic sites and its total acidity was estimated to be 6.3 mmol/g.cat. To further validate this assumption, the Brønsted acidity of both CS/MIL-53(Al) and CS/MIL-53(Al)-SO_3_H was compared via UV–Vis spectroscopy and Hammett equation (Eq. 1).

1 $${\text{H}}^\circ = {\text{ p}}K\left( {\text{I}} \right)_{{{\text{aq}}.}} + {\text{ log }}\left[ {\text{I}} \right] \, /\left[ {{\text{IH}}} \right]^{ + }$$

where p*K*(I) is the pK_a_ value for I.

According to this method, UV–Vis spectrum of a basic indicator [I], such as 4-nitroaniline is obtained at λ_max_ = 382 nm. Then, UV–Vis spectrum of I in the presence of each catalytic sample, i.e. CS/MIL-53(Al) or CS/MIL-53(Al)-SO_3_H was also recorded, Figure S1. As in the presence of the acidic species, I will be protonated to form [IH]^+^, it is expected that the absorbance will decrease by increase of the acidity. Having the adsorption of [I] and [IH]^+^, Hammett function, H°, is simply calculated, Eq. (1). According to the results, Table S1, H° value for CS/MIL-53(Al)-SO_3_H is almost half of CS/MIL-53(Al), indicating that sulfonation remarkably increased the acidity of the composite. To further confirm this issue, the catalytic activity of the both samples for dehydration of fructose to HMF at 110 °C for 40 min was measured. Gratifyingly, it was observed that the yield of HMF in the presence of CS/MIL-53(Al)-SO_3_H was 97.1%, which was superior compared to CS/MIL-53(Al), 30%. This finding indicates the role of sulfonation in the catalytic activity of the composite.

### Catalytic conversion of fructose to HMF

#### Optimization of the reaction

Several experiments were carried out according to the similar reported catalysts^52^ before the investigation of the RSM statistical method which is well-established as a potent tool for assessing the synergism among the reaction parameters (Table S2). To end this, the impact of reaction time on the yield of HMF from fructose dehydration was investigated using CS/MIL-53(Al)-SO_3_H catalyst (40 W%) in DMSO (1.5 mL) at 100 °C as a model reaction. The findings, presented in Table S2, indicated that a yield of 60% was achieved within 30 min. Prolonging the reaction time resulted in an increase in HMF yield up to 69%. Furthermore, determining the effect of catalyst amount on the HMF yield revealed that the HMF yield increased from 45 to 69% as the catalyst loading and the availability of acid sites increased from 20 to 40 W%. Lastly, the relationship between reaction temperature and the conversion of fructose to HMF was examined. It was observed that increasing the temperature from 90 °C to 110 °C further enhance the yield of HMF from 55 to 95%, respectively. As a result, using 30 W% of the catalyst at 110 °C in 40 min for every 50 mg of fructose was considered sufficient for obtaining the highest HMF yield (95%).

However, for the accurately optimization of three main parameters, reaction time, temperature and CS/MIL-53(Al)-SO_3_H loading, in the fructose dehydration process, RSM method was utilized. Table S3 summarizes the results of a quadratic model analysis of variance (ANOVA) of the data obtained on the impact of the aforesaid parameters during the synthesis of HMF. According to the result, the synergism among the studied factors can be declared as follow (Eq. 2):

2 $${\mathbf{Yield}} \, {\mathbf{of}} \, {\mathbf{HMF}} \, \left( \% \right) = \, + {95}.{23} + {2}.{\text{81A}} - {3}.{\text{31B}} + {5}.{\text{81C}} - {5}.{\text{63AB}} - {5}.{\text{62AC}} + {7}.{\text{88BC}} - {9}.{\text{89A}}^{{2}} - {12}.0{\text{1B}}^{{2}} - {13}.{\text{64C}}^{{2}}$$

In Eq. (2), the positive coefficients of A, C and BC indicate the synergistic effects of them in HMF synthesis. Conversely, the negative coefficients of B, AB, AC, A^2^, B^2^, and C^2^ implied their antagonistic effect on HMF production. Considering the coefficients, it can also be concluded that the effects of the parameters follow the order of C^2^ > B^2^ > A^2^ > BC > C > AB > AC > B > A.

The values of the correlation coefficient, R^2^, the Adjusted R^2^ and the Predicted R^2^ were 0.96, 0.92 and 0.77 respectively, confirming the accuracy of the model. Optimization of the reaction conditions using RSM indicated that reaction in the presence of a catalyst (23 wt%) in DMSO, at 110 °C for 40 min led to the formation of HMF in 97.1% (Figures S3 and S4).

**Figure 5:**
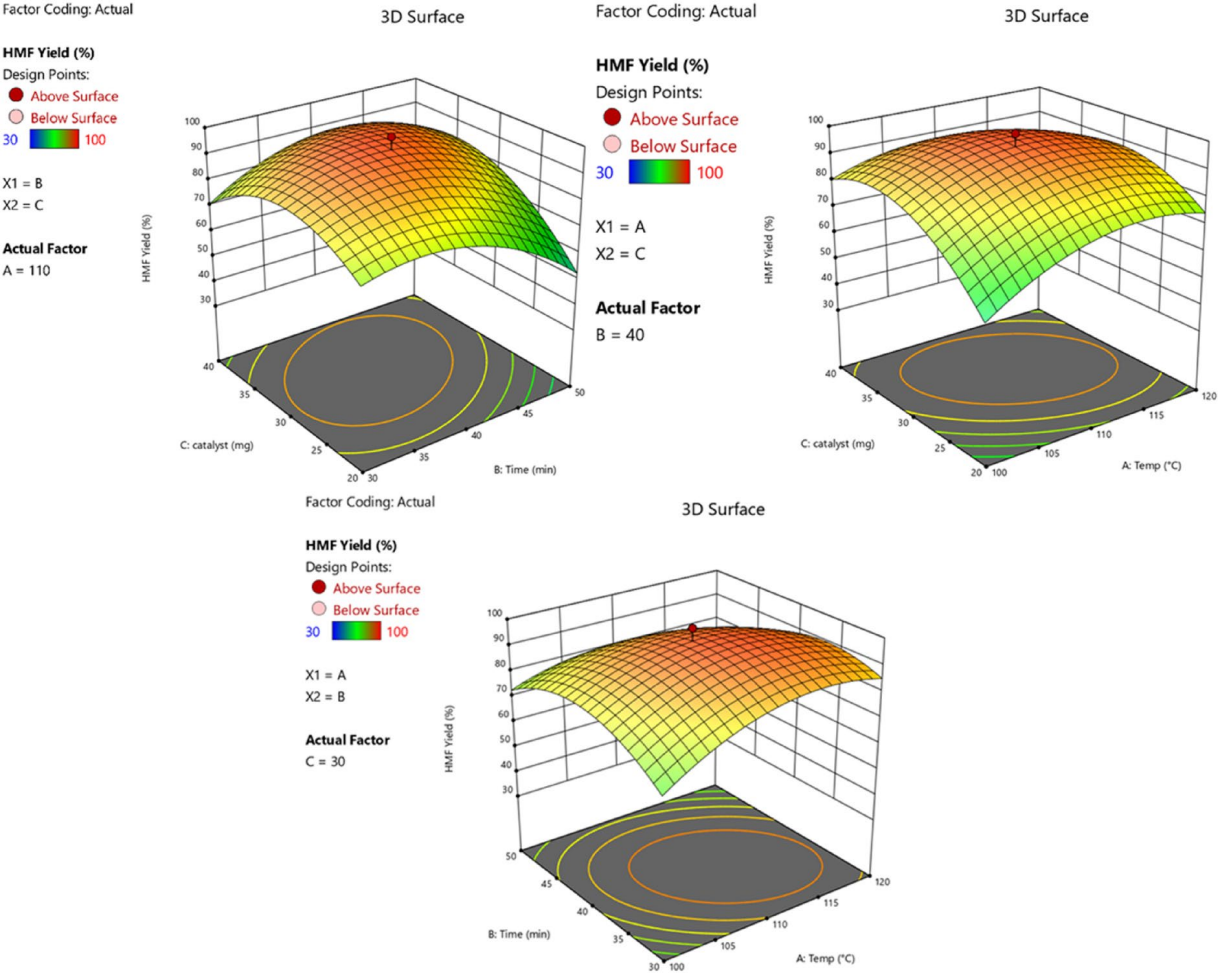
The three-dimensional (3D) plots of the effect of reaction temperature, time, and catalyst amount on the yield of the HMF product.

The 3D surface plots of the interactions amongst the understudied parameters as a function of HMF yield are presented in Fig. 5. Considering these plots, it is concluded that upon prolonging the reaction to 40 min, the HMF yield constantly increased. However, continuing the reaction for a longer time led to a decrease in HMF yield, which can be a result of formation of by-products and condensation of HMF to humin. Moreover, it can be seen that increase of CS/MIL-53(Al)-SO_3_H loading, which means access to more active catalytic sites, led to the improvement of HMF yield. However, there is an optimum value (23 wt%) for this parameter and use of more catalysts resulted in a detrimental effect on HMF yield. In fact, by increasing the catalyst loading, more acidic sites will be available, which may promote the formation of humin or/and by-products. Finally, RSM results showed that reaction temperature is also an effective parameter on the HMF production and its optimum value was 110 °C.

#### Mechanism of fructose dehydration

**Figure 6:**
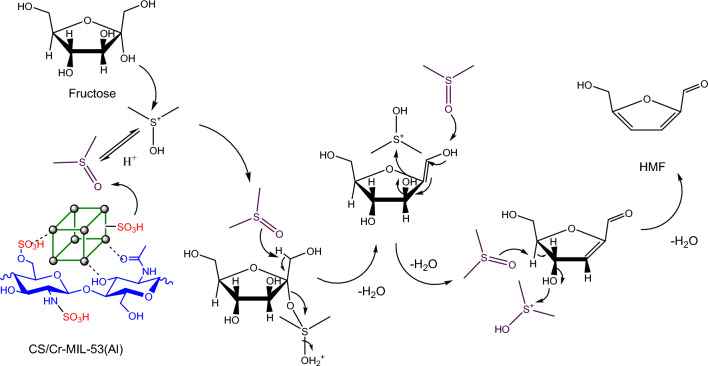
Possible mechanism for acid-catalyzed dehydration of fructose to HMF.

The plausible fructose dehydration mechanism using CS/MIL-53(Al)-SO_3_H in DMSO is illustrated in Fig. 6. According to the literature^65^, DMSO as a polar solvent can contribute to the reaction. More precisely, in the first step of the reaction, proton is transferred from CS/MIL-53(Al)-SO_3_H to DMSO. Subsequently, the -OH group on fructose attacks the electrophilic (S atom) center of protonated DMSO, leading to the formation of O-S bond. Afterward, proton shift from –OH functionality of anomeric center to O atom in DMSO occurs. Finally, the involvement of other DMSO molecules and repeating of the process, followed by removal of three H_2_O molecules results in the formation of HMF.

#### Recyclability test

**Figure 7:**
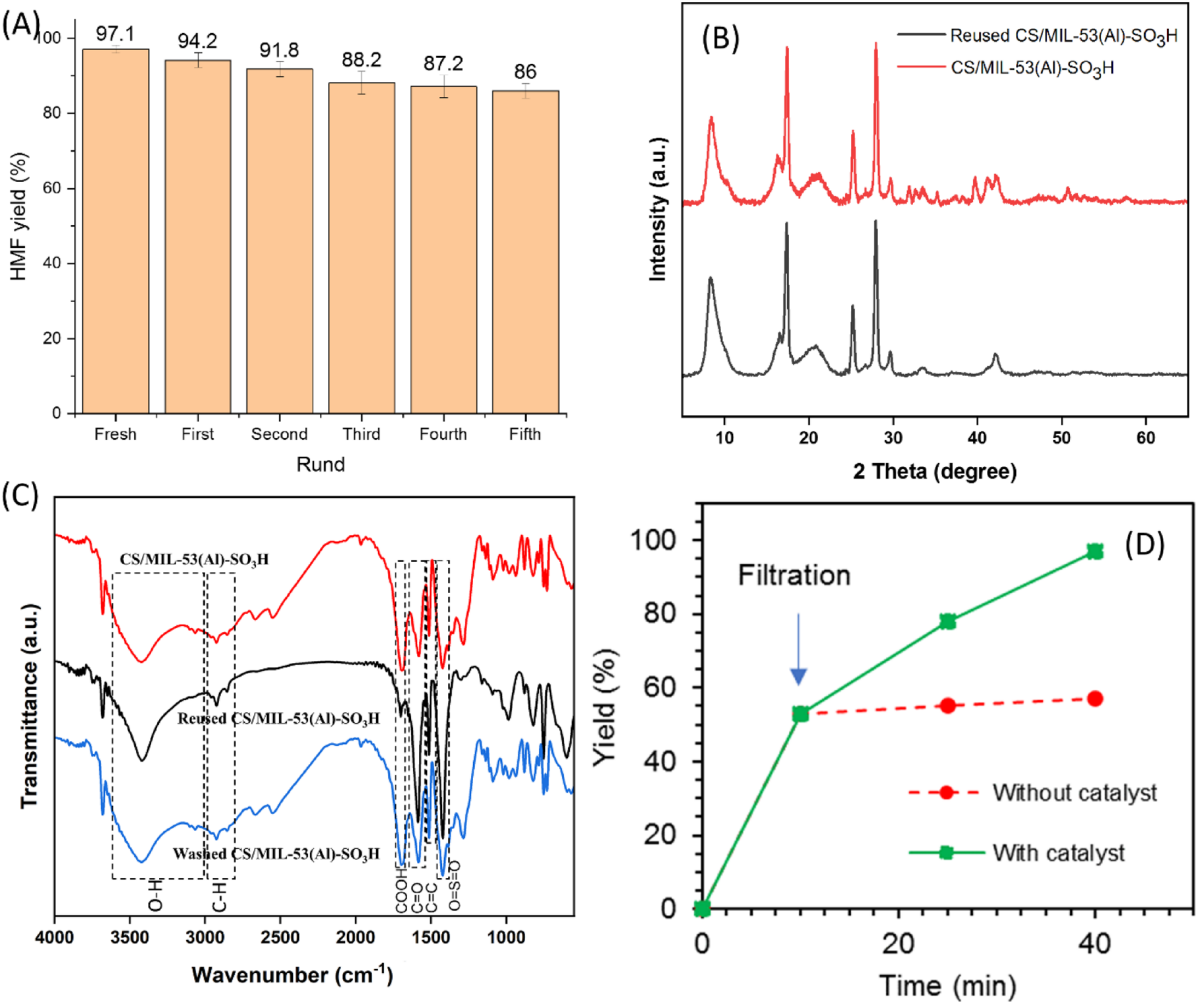
(**A**) the recyclability of CS/MIL-53(Al)-SO_3_H catalyst, conditions: fructose (50 mg), catalyst (23 wt%), DMSO (1 mL), at 110 °C for 40 min. (**B**) Comparison of XRD patterns and (**C**) FTIR spectra of fresh and reused catalyst. (**D**) Hot filtration tests of CS/MIL-53(Al)-SO_3_H catalyst.

To evaluate the recyclability of CS/MIL-53(Al)-SO_3_H, the conventional recycling test was conducted for dehydration of fructose under the optimal conditions for five consecutive runs. As described in the Experimental section, the separated CS/MIL-53(Al)-SO_3_H was rinsed with DMSO repeatedly to wash the possible deposited products and substrate from the catalyst surface and dried at room temperature overnight. Measuring the yield of each dehydration run, Fig. 7A, established that recycling of CS/MIL-53(Al)-SO_3_H did not cause considerable loss of its activity, confirming high recyclability of CS/MIL-53(Al)-SO_3_H. Motivated by this result and with the aim of elucidating the stability of the catalyst, the recovered CS/MIL-53(Al)-SO_3_H after the last run of the reaction was characterized via FTIR and XRD. As depicted in Fig. 7B, the XRD pattern of the reused CS/MIL-53(Al)-SO_3_H was exactly similar to the fresh one and no displacement of the characteristic peaks was observed. This finding emphasizes the structural stability of CS/MIL-53(Al)-SO_3_H upon recycling. Similarly, the FTIR spectrum of the reused CS/MIL-53(Al)-SO_3_H, Fig. 7C, showed the same characteristic absorbance bands detected in the fresh catalyst, confirming chemical stability of the catalyst. Notably, the band at 1703 cm^−1^ attributed to –COOH functionality was diminished and some new absorbance bands appeared in the FTIR spectrum of the reused CS/MIL-53(Al)-SO_3_H. These can be due to the deposition of organic components on the surface of the catalyst through interacting with –COOH functionality as the related band was relatively decreased. This issue, i.e. coverage of some active sites of CS/MIL-53(Al)-SO_3_H, can be the origin of decrement of the catalytic activity upon recycling. To resolve this problem, the reused catalyst was washed several times with DMSO, which showed similar spectrum than that of fresh catalyst (Fig. 7C). To further investigate this issue, the Brønsted acidity of reused CS/MIL-53(Al)-SO_3_H was measured and compared with that of the fresh one, Table S1. As listed, the acidity of the reused catalyst was slightly lower than that of fresh CS/MIL-53(Al)-SO_3_H, indicating that the catalyst preserved the majority of its active site in the course of the reaction. In fact, as –SO_3_H groups have been introduced covalently on the CS/MIL-53(Al) nanocomposite, they will remain on the catalyst upon recycling. Moreover, the acid–base back titration of CS/MIL-53(Al)-SO_3_H and reused CS/MIL-53(Al)-SO_3_H determined to be 0.40 ± 0.01 mmol/g and 0.39 ± 0.01 mmol/g, respectively. Furthermore, the hot filtration test was conducted to verify the heterogeneous nature of catalysis. In more detail, dehydration of fructose to HMF was halted after a short time and then CS/MIL-53(Al)-SO_3_H was separated from the reaction media. Monitoring of the yield of the reaction after catalyst removal and its comparison with the reaction in the presence of the catalyst, Fig. 7D, indicated that upon removal of the catalyst, no remarkable HMF yield improvement was observed, which indicated the true heterogeneous nature of the catalysis.

#### Comparative study

As discussed, CS/MIL-53(Al)-SO_3_H exhibited high catalytic activity and recyclability for dehydration of fructose to HMF. To further shed light on the performance of this bio-based catalyst, its activity was compared with some other catalysts, which have been reported previously. Obviously, the difference in the nature of the catalysts and reaction conditions does not allow an accurate comparison and this comparison is just a random study to establish whether CS/MIL-53(Al)-SO_3_H can be deemed as a potential catalyst for the synthesis of HMF. As tabulated in Table 1, entry, some catalysts, such as MIL-101(Cr) and MIL-53(Al)-SO_3_H were not efficient for the synthesis of HMF. On the other hand, some efficient catalysts, such as SO_3_H-dendrimer-SiO_2_@Fe_3_O_4_ require a multi-step synthetic procedure, which makes their synthesis tedious. Heterogeneous heteropolyacid-based catalysts, such as halloysite-supported Keggin, Hal-SiW, have also been reported recently. Although the catalytic activity of this catalyst is high, it is inferior compared to that of CS/MIL-53(Al)-SO_3_H. Moreover, handling heteropolyacids, which are highly soluble is challenging. CS/MIL-53(Al)-SO_3_H catalyst, however, can be synthesized through a relatively facile protocol using bio-based CS, which is naturally available. In the sulfonation of CS/MIL-53(Al), HSO_3_Cl not only can be reacted with terephthalic acid ligand of MIL-53(Al), but also amine functional groups of CS participated in this reaction toward synthesis of highly functionalized Bronsted said-functionalized catalyst (Fig. 1). Therefore, its performance is comparable to or higher than some other reported catalysts, with no need for any co-catalyst or ionic liquid. All these factors render CS/MIL-53(Al)-SO_3_H catalyst a potential catalyst for fructose dehydration to HMF.

**Table 1:** Comparison of the catalytic activity of CS/MIL-53(Al)-SO_3_H catalyst for HMF production from fructose substrate with reported solid acid catalysts.

Entry	Catalyst	Solvent	Temp. (°C)	Time (min)	HMF yield (%)	References
1	SO_3_H-dendrimer-SiO_2_@Fe_3_O_4_	–	100	60	92	^66^
2	SBA-15-SO_3_H	Ionic liquid	120	60	81	^67^
3	Fe_3_O_4_@SiO_2_-SO_3_H	DMSO	100	120	93.1	^68^
4	4KSCC^a^	Organic solvent/water	140	40	80.9	^69^
5	Hal-SiW^b^	DMSO	125	43	99.5	^70^
6	Hal/*k*-Cr/PAA^c^	DMSO	100	35	97.9	^71^
7	MIL-101(Cr)	DMSO	120	60	24	^52^
8	MIL-101(Cr)-SO_3_H	DMSO	120	60	90	^52^
9	MIL-53(Al)-SO_3_H	DMSO	120	60	79	^52^
10	CS/MIL-53(Al)-SO_3_H	DMSO	110	40	97	This work

^a^Sulfonated chitosan-derived carbon-based catalyst.

^b^Keggin-type heteropolyacids impregnated upon halloysite.

^c^Halloysite/*k*-Carrageenan/polyacrylic acid nanocomposite.

## Experimental

### Materials

Chitosan (CS, obtained from shrimp shells, ≥ 75% deacetylated) and chlorosulfuric acid (HSO_3_Cl) (99.8% purity) were purchased from Sigma-Aldrich Co. Aluminium nitrate nonahydrate (Al(NO_3_)_3_.9H_2_O) (≥ 98% purity), terephthalic acid (H_2_BDC, 98% purity), acetic acid (≥ 99% purity) and fructose (> 99% purity) were obtained from Merck KGaA Co. Dichloromethane (≥ 99.8% purity), acetone (2-propanone or dimethyl ketone) (≥ 99.5% purity) and DMSO (≥ 99.9% purity) were provided from CARLO ERBA Reagents (S.A.S) and ethanol (EtOH) (99.8% purity) was obtained from Kimia Alcohol Zanjan Co. (PJS).

### Synthesis of CS/MIL-53(Al)

To prepare CS/MIL-53(Al) nanocomposite, the reported procedure at room temperature using a precipitation technique for the synthesis of MIL-53(Al) was used with some modifications^57^. Briefly, a solution of 665 mg (4 mmol) of H_2_BDC and 320 mg (8 mmol) NaOH in 10 mL of H_2_O was added in a dropwise manner to an as-prepared solution formed by dissolving 200 mg of chitosan in 10 mL distilled water containing 100 μL of acetic acid and 1600 mg (80 mmol) of Al(NO_3_)_3_·9H_2_O. Such addition makes the immediate appearance of a white precipitate. The mixture was maintained at room temperature for one week. The white precipitate of any aliquot was washed with distilled water and ethanol repeatedly and dried at room temperature overnight.

### Synthesis of CS/MIL-53(Al)-SO_3_H

According to the reported method^52^, to functionalize the synthesized CS/MIL-53(Al) with sulfonic acid, 553 mg of CS/MIL-53(Al) was dispersed in 17 mL of dichloromethane under a temperature of 0 °C for 20 min. Then, HSO_3_Cl (0.16 mL in 6 mL, 0.14 M) was added gradually to the above mixture under vigorous stirring. After 30 min, the obtained solid product was centrifuged at 8400 rpm, then washed with distilled water and acetone. To purify CS/MIL-53(Al)-SO_3_H, it was dispersed in ethanol and treated under reflux conditions for 24 h. Subsequently, the solid was centrifuged at 8400 rpm, rinsed several times with ethanol, and dried in the oven at 120 °C, Fig. 1.

### Characterization of CS/MIL-53(Al)-SO_3_H

PANalytical (XPert Pro MPD) apparatus with Cu and K = 1.54060 nm was applied for recording the X-ray diffraction (XRD) patterns of CS, CS/MIL-53(Al), and the fresh and reused catalysts. Thermo gravimetric analysis (TGA) of CS, CS/MIL-53(Al) and CS/MIL-53(Al)-SO_3_H was conducted by Mettler Toledo thermal calorimeter in which, the heating was carried out under an oxygen atmosphere with a temperature increase of 10 °C per minute. The morphology of CS/MIL-53(Al)-SO_3_H was appraised by Scanning electron microscopy (SEM) using VEGAII TESCAN scanning electron microscope, equipped with QX2, RONTEC energy dispersive X-ray analyzer. X-ray photoelectron spectroscopy (XPS) analysis was done using a Bes Tek instrument with Al being as the X-ray anode at a vacuum pressure of -10 mbar and Kα equal to 1486.6 eV. Fourier transform infrared spectrometry (FTIR; Perkin Elmer, spectrum RX I) was employed to identify the chemical bonding of CS, CS/MIL-53(Al), and fresh and reused CS/MIL-53(Al)-SO_3_H. The Brønsted acidity of CS/MIL-53(Al) and fresh and reused CS/MIL-53(Al)-SO_3_H was determined using UV–Vis spectroscopy and the Hammett equation, vide infra. Nitrogen adsorption–desorption analysis was carried out at 77 K using BELSORP-mini II (Japanese). The acidity of CS/MIL-53(Al)-SO_3_H was studied by the temperature-programmed desorption of ammonia (NH_3_-TPD(, Chemisorption Analyzer, NanoSORD (made by Sensiran Co., Iran), the heating ramp rate was 20 °C/min, in the temperature range of 25–800 °C).

### General procedure for fructose conversion into HMF

First, fructose (50 mg) was dissolved in DMSO and then mixed with desired amount of CS/MIL-53(Al)-SO_3_H under stirring conditions. The reaction was continued at 300 rpm at chosen temperature and duration time. Afterward, the mixture was centrifuged at 8400 rpm for 3 min to separate CS/MIL-53(Al)-SO_3_H, which was then washed several times with DMSO and dried at room temperature overnight for reusing.

## Conclusion

This study focused on creating a new composite material called CS/MIL-53(Al)-SO_3_H by combining sulfonated CS and MOF. The composite was made by forming MIL-53(Al) in the presence of CS and then treating it with chlorosulfuric acid. The composite was characterized and tested as a catalyst for converting fructose to HMF through dehydration. Various characterization techniques were performed that validated the successful formation of the CS/MIL-53(Al)-SO_3_H composite. By optimizing the reaction conditions using RSM, it was found that using 23% catalyst in DMSO at 110 °C for 40 min resulted in a 97.1% yield of HMF. The sulfonation of the CS/MIL-53(Al) composite was found to significantly affect its acidity and catalytic performance. On the other hand, the designed catalyst based on CS biopolymer showed remarkable performance in fructose dehydration, owing to its accessible sulfonic acid groups and coordinatively unsaturated Al^3+^ sites. Additionally, the CS/MIL-53(Al)-SO_3_H composite could be reused for five runs with only an 11.1% decrease in yield. The present composite stands out as an environmentally friendly and efficient catalyst with promising applications, thanks to its straightforward synthesis, use of bio-derived and affordable initial components, remarkable catalytic performance, and recyclability.

### Supplementary Information

Supplementary Information.

## Data Availability

The data that support the findings of this study are available from the corresponding author, upon reasonable request.
