# Supplementary material for: Sulfonic acid-functionalized chitosan–metal–organic framework composite for efficient and rapid conversion of fructose to 5-hydroxymethylfurfural
Source: Sci Rep. 2024 Mar 10;14:5834. doi: 10.1038/s41598-024-56592-3 (PMC10925054; doi:10.1038/s41598-024-56592-3)
Supplement: Supplementary file 1 — Supplementary Information. [file 41598_2024_56592_MOESM1_ESM.docx]

**Supporting information**

**Sulfonic acid-functionalized chitosan-metal-organic framework composite for efficient and rapid conversion of fructose to 5-hydroxymethylfurfural**

Sima Darvishi^1^, Samahe Sadjadi^2^^[[1]](#footnote-1)^, Majid M. Heravi^1^

*^1^Department of Chemistry, School of physic and chemistry, Alzahra University, PO Box 1993891176, Vanak, Tehran, Iran.*

*^2^Gas Conversion Department, Faculty of Petrochemicals, Iran polymer and Petrochemical Institute, PO Box 14975-112, Tehran, Iran. Email: s.sadjadi@ippi.ac.ir*


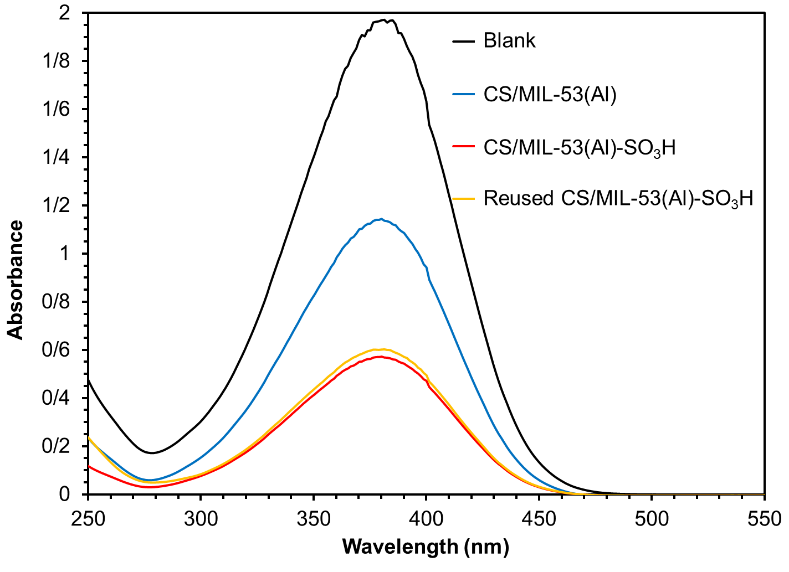


**Fig. S1.** Hammett plot for the catalysts using the basic 4-nitroanilin indicator.

Moreover, the investigation involved the examination of the conversion of various sugars using the CS/MIL-53(Al)-SO_3_H catalyst (Fig. S2). Notably, fructose demonstrated the highest HMF yield among these sugars. It is essential to note that, under the optimized conditions, the conversion of glucose, cellulose, galactose, and sucrose to HMF requires two or three principal steps: hydrolysis, isomerization, and dehydration into HMF. Consequently, the efficiency of their conversion to HMF is lower compared to fructose.


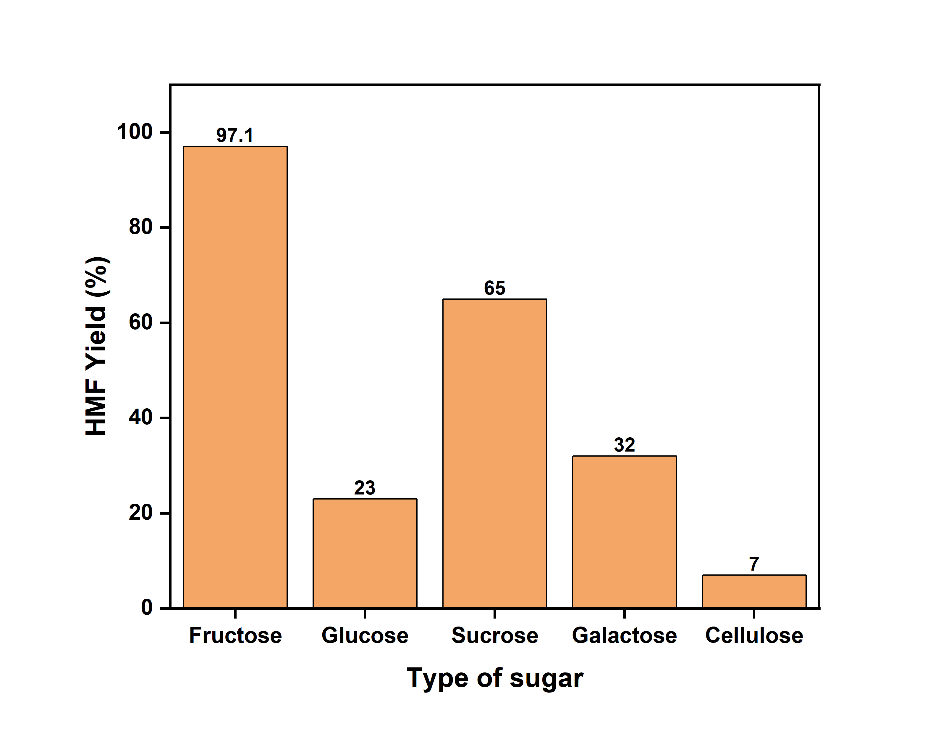


**Fig. S2.** Conversion of different sugars to HMF using CS/MIL-53(Al)-SO_3_H catalyst performed in DMSO under the optimized reaction conditions.

**Table S1.** Values of Hammett function (H^o^)

| H^o^ | [IH]% | [I]% | A_max_^α^ | Catalyst | Entry |
| --- | --- | --- | --- | --- | --- |
| - | - | 100 | 1.969 | Blank | 1 |
| 1.077 | 46.161 | 53.839 | 1.123 | CS/MIL-53(Al) | 2 |
| 0.594 | 72.260 | 27.740 | 0.644 | CS/MIL-53(Al)-SO_3_H | 3 |
| 0.617 | 71.187 | 28.813 | 0.630 | Reused CS/MIL-53(Al)-SO_3_H | 4 |
| ^α^ Indicator: 4-nitroaniline | | | | | |

**Table S2.** Single-factor experiments for the fructose conversion into HMF

| HMF Yield% | Time (min) | Catalyst (W%) | Temp (°C) | Run |
| --- | --- | --- | --- | --- |
| 55 | 40 | 30 | 90 | 1 |
| 45 | 30 | 20 | 100 | 2 |
| 60 | 30 | 40 | 100 | 3 |
| 69 | 50 | 40 | 100 | 4 |
| 95 | 40 | 30 | 110 | 5 |
| 53 | 20 | 30 | 110 | 6 |

**Table S3.** The ANOVA results of the response surface reduced quadratic model.

| Source | Sum of Squares | df | Mean Square | F-value | p-value |  |
| --- | --- | --- | --- | --- | --- | --- |
| Model | 9479.07 | 9 | 1053.23 | 26.86 | < 0.0001 | significant |
| A-Tem | 126.56 | 1 | 126.56 | 3.23 | 0.1026 |  |
| B-Time | 175.56 | 1 | 175.56 | 4.48 | 0.0604 |  |
| C-catalyst | 540.56 | 1 | 540.56 | 13.79 | 0.0040 |  |
| AB | 253.13 | 1 | 253.13 | 6.46 | 0.0293 |  |
| AC | 253.13 | 1 | 253.13 | 6.46 | 0.0293 |  |
| BC | 496.13 | 1 | 496.13 | 12.65 | 0.0052 |  |
| A² | 2457.47 | 1 | 2457.47 | 62.67 | < 0.0001 |  |
| B² | 3627.43 | 1 | 3627.43 | 92.51 | < 0.0001 |  |
| C² | 4675.32 | 1 | 4675.32 | 119.23 | < 0.0001 |  |
| Residual | 392.13 | 10 | 39.21 |  |  |  |
| Lack of Fit | 271.30 | 5 | 54.26 | 2.25 | 0.1977 | not significant |
| Pure Error | 120.83 | 5 | 24.17 |  |  |  |
| Cor Total | 9871.20 | 19 |  |  |  |  |

**^1^HNMR of HMF**

^1^HNMR analysis was carried out to consider the structure of the extracted HMF from the catalytic reaction mixture. As seen in Figure S1, the ^1^HNMR spectrum of the as-synthesized HMF displays the peaks at δ 4.50 (d, 2H, CH_2_), δ 5.62 (t, 1H, OH), δ 6.60 (d, 1H, furan ring C-c proton), δ 7.49 (d, 1H, furan ring C-d proton), and δ 9.54 (s, 1H, CHO), confirming successful conversion of HMF.^1,2^ Notably, the peaks appeared at δ 2.5 and 3.4 are ascribed to the solvent and its impurities (water) respectively.^3^


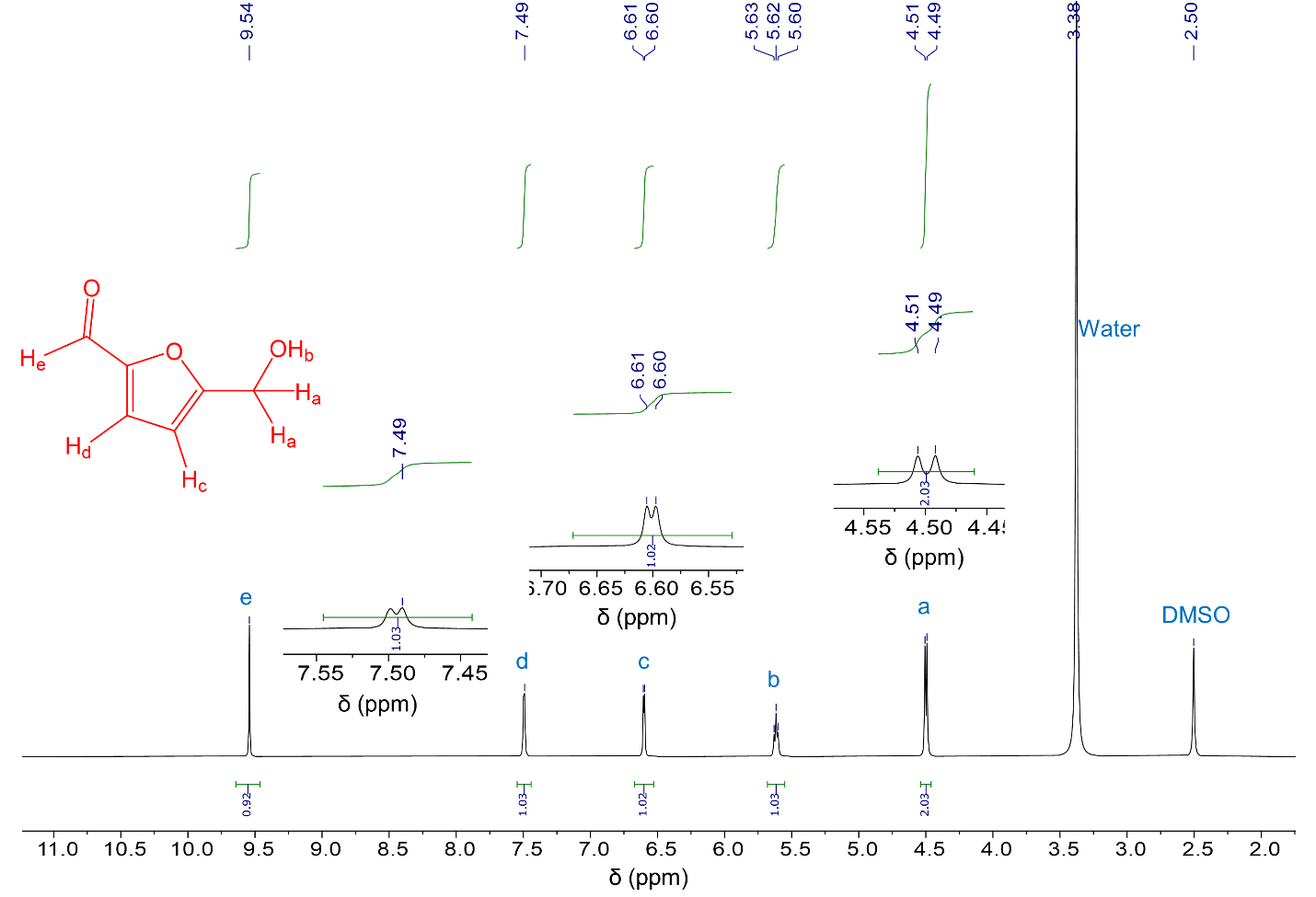


**Figure S3.** ^1^HNMR spectrum of the as-synthesized HMF


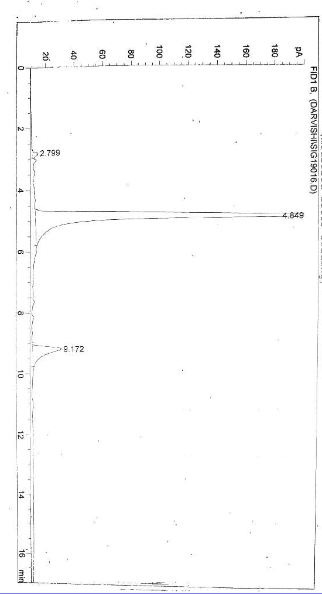


**Figure S4.** GC Spectrum of the supernatant of catalyst under optimization conditions: catalyst (23 wt%) in DMSO, at 110 °C for 40 min.

**Fig. S5.** NH_3_-TPD curve of the CS/MIL-53(Al)-SO_3_H.

**References**

1 Wang, J., Ren, J., Liu, X., Lu, G. & Wang, Y. High yield production and purification of 5‐hydroxymethylfurfural. *AICHE J.* **59**, 2558-2566 (2013).

2 Serra-Cayuela, A. *et al.* Identification of 5-hydroxymethyl-2-furfural (5-HMF) in Cava sparkling wines by LC-DAD-MS/MS and NMR spectrometry. *Food Chemistry* **141**, 3373-3380, doi:<https://doi.org/10.1016/j.foodchem.2013.05.158> (2013).

3 Sadjadi, S., Yaghoubi, S., Zhong, X., Yuan, P. & Heravi, M. M. Tuning the acidity of halloysite by polyionic liquid to develop an efficient catalyst for the conversion of fructose to 5-hydroxymethylfurfural. *Sci. Rep.* **13**, 7663 (2023).

1. Corresponding author: *Tel: +98 2148666; Fax: +98 214478, Email:* [*s.sadjadi@ippi.ac.ir*](mailto:s.sadjadi@ippi.ac.ir) [↑](#footnote-ref-1)
